# An integrated individual, community, and structural intervention to reduce HIV/STI risks among female sex workers in China

**DOI:** 10.1186/1471-2458-13-717

**Published:** 2013-08-04

**Authors:** Dianmin Kang, Xiaorun Tao, Meizhen Liao, Jianzhuo Li, Na Zhang, Xiaoyan Zhu, Xiaoguang Sun, Bin Lin, Shengli Su, Lianzheng Hao, Yujiang Jia

**Affiliations:** 1 Institution for AIDS/STD Control and Prevention & Shandong Key Laboratory for Epidemic Disease Control and Prevention, Shandong CDC, Jinan, Shandong Province 250014, P. R. China; 2 Institute for AIDS/STD Control and Prevention, Jinan Municipal Center for Disease Control and Prevention, Jinan, Shandong Province 250001, P. R. China; 3 Department of Preventive Medicine, Vanderbilt University, Nashville, TN 37232, USA

**Keywords:** Female sex workers, HIV, Condom use, Intervention counties, China

## Abstract

**Background:**

We assessed the effectiveness of an integrated individual, community, and structural intervention to reduce risks of HIV and sexually transmitted infections (STIs) among female sex workers (FSWs).

**Methods:**

The integration individual, community, and structural intervention was implemented from 2004 to 2009 in six counties of Shandong Province. Post-intervention cross-sectional surveys were conducted in six intervention counties and 10 control counties.

**Results:**

Of 3326 female sex workers were recruited and analyzed in the post-intervention survey with 1157 from intervention sites and 2169 from control sites. No HIV positive was found in both intervention and control counties. The rate of syphilis was 0.17% for intervention sites and 1.89% for control sites (OR = 11.1, 95% CI: 2.7, 46.1). After adjusted for age, marital status, education, economic condition, recruitment venues, the rates of condom use in the last sex with clients(AOR = 2.7; 95% CI: 1.9, 3.8), with regular sex partners(AOR = 1.5; 95% CI: 1.1, 1.9) and consistent condom use in the last month with clients (AOR = 3.3; 95% CI: 2.6, 4.1) and regular sex partners (AOR = 1.7; 95% CI: 1.3, 2.3) were significantly higher in intervention sites than that in control sites. The proportion of participants correctly answered at least six out of eight HIV-related questions (83.3%) in intervention sites is significant higher than that (21.9%) in control sites (AOR = 24.7; 95% CI: 2.5, 42.7), the five indicators related to HIV-related intervention services ever received in the last year including HIV testing(AOR = 4.9; 95% CI: 2.8, 6.7), STD examination and/or treatment(AOR = 5.1; 95% CI: 4.2, 6.4), free condom(AOR = 20.3; 95% CI: 14.3, 28.9), peer education(AOR = 4.3; 95% CI: 3.5, 5.4), education materials(AOR = 19.8; 95%CI: 13.1, 29.8) were significantly higher in intervention sites than that in control sites, the participants in the intervention sites are more likely to seek medical treatment when they had any disorders (AOR = 3.2; 95% CI: 2.5, 4.2).

**Conclusion:**

This study found that the integrated individual, community, and structural intervention showed positive impact in reducing HIV and STI risks among FSWs.

## Background

Efforts in HIV risk reduction for female sex workers (FSWs) have often relied on not only individual- level interventions, e.g., condom promotion and sexually transmitted infection (STI) management [1-4], but also structural interventions to alter the social and physical environments in which individual behave [5,6]. Two structural interventions have been applied in the context of sex work – community mobilization initiatives such as the “Sonagachi project” in India [7,8] and government policy initiatives such as the Thai “100% condom program” [9,10] – and program assessments of each have documented decreases in unprotected sex and prevalence of STI. Some countries have sought to adopt or adapt either the community mobilization [11,12] or government policy approach [13,14] to prevent HIV and STI among FSWs and their partners. The integrated interventions with both community mobilization and government policy demonstrated positive effects on HIV and STI risk reduction among FSWs [15]. Structural factors, including political, legal, social, cultural, economic, and environmental factors, are known to affect individual risk and vulnerability to HIV, and to operate at several different societal levels (individual, interpersonal, community, culture and policy) [16,17].

Sexually transmitted diseases (STDs) were nearly eliminated in China after the new government was established in 1950’ [18]. Structural interventions played a critical role in the process, e.g., the establishment of infrastructure of health facilities in all workplace, training and dispersion of the “barefoot doctors”, elimination of prostitution, emancipation of women, full employment, education campaign, and expanded public health intervention for screening and treatment of syphilis and other STI [19,20]. However, following the economic reforms which began in the 1980s, STDs reappeared and today constitute a major public health problem in China. This resurgence parallels the privatization of the economy, the breakdown of the national health insurance system, the return of prostitution, and other factors. The commercial sex was driven and reinforced following the growing economic disparities, population migration, increased disposable income, changing in sexual attitude, reestablishment and growth of sex trade [21-26], that fueled the resurgence of STDs in the nation [22]. As the most populous country in the world, today China confronted with the huge challenges that HIV and STI pose. Injection drug use has been the predominant route of the transmission for HIV in the early stage of the epidemic [27,28], however, two thirds of national estimated 78,000 HIV/AIDS cases in 2011 were infected through sexual transmission with a half through heterosexual contact [29]. The experience of African countries has already indicated that when the heterosexual transmission became the main mode of HIV transmission, the epidemic grew rapidly. Hence, heterosexual transmission of HIV through unprotected contact with FSWs is of particular concern in China [30].

China initiated a series of intervention programs among high-risk populations in the 1990's, including providing standardized STI testing and treatment for FSWs during police crackdowns on prostitution [31]. The Global Fund AIDS Program was implemented in certain areas in China in 2004, including six counties in Shandong Province. Along the east coast, Shandong is the second most populous provinces in China. The first HIV case in Shandong was identified in 1992; there were 3740 cumulative reported HIV/AIDS cases through the end of 2012. The proportion of heterosexual contact has been increasing in the newly reported HIV/AIDS cases in Shandong Province, nearly half in 2011 [32]. Both HIV and syphilis prevalence rates in the general population in Shandong province remains at relatively low level. Data from the sentinel surveillance in Shandong showed that the HIV and syphilis prevalence among the general population was 0.03% and 0.20% respectively in 2011, but it increases rapidly among some specific groups in the recent years [33]. With the support from the Global Fund AIDS Program, six intervention counties implemented an integrated individual, community, and structural intervention among FSWs from 2004 to 2009. This study applied post intervention cross-sectional surveys to investigate the impact of the integrated interventions among FSWs.

## Methods

### Study design

The integrated individual, community, structural intervention program among FSWs was implemented from 2004 to 2009 in six intervention counties (Xintai, Caoxian, Chengwu, Mudanqv, Pingdu, and Zhoucheng) supported by the China’s Global Fund AIDS program. The integrated individual level intervention, community mobilization and structural interventions included community solidarity and collective commitment, multisectoral government community partnership coordination, stigma and discrimination reduction at all settings, 100% condom promotion activities, outreach and peer education to promote risk and health-seeking behavior change, expand HIV testing and promote standardization of clinical STI services, monitoring and encouraging adherence, improvement of the access to high quality of HIV/AIDS treatment, care and support and secondary prevention services for people living with HIV [34,35]. The selection of these six intervention sites were based on the number of HIV/AIDS case reporting among total 140 counties of Shandong. Xintai and Pingdu are developed sites economically and the rest four are less developed sites. Ten control counties were selected including four developed counties (Huaiyin, Longkou, Rushan, Zhiwu) and six less developed counties (Decheng, Dongming, Gaomi, Jvxian, Pingyin, Yanggu). The cut of value between economically developed and less developed counties was $2500 of average per capita income of its residents. The selection of control sites was to ensure that the background characteristics between intervention and control sites are similar in the baseline including the sociodemographics, economical development, and the status of HIV/STI epidemics. The six intervention sites hosted 7.2 million with 103 cases of HIV/AIDS reported through the year of 2004 compared with 10.9 million population and 32 diagnosed and reported cases of HIV/AIDS in the 10 control sites.

During the intervention period from 2004 to 2009, the integrated individual, community, structural intervention supported by the Global Fund AIDS Program was only implemented in the six intervention sites as described in the following “Intervention components”. The ten control sites and all other counties or cities across the province were provided the “standard of care”, the routine testing, prevention, care and treatment services for HIV and STI, but not the integrated individual, community, and structural intervention.

### Intervention components

#### ***Leadership and governance, community solidarity and collective commitment***

To support the effective implementation of the integrated individual, community, structural intervention program, the Global Fund AIDS Program committees were established and chaired by the senior government officials, and comprised the heads of all government development departments, including health, police, legislation, propaganda, education, finance, labor, commerce, civil, construction and engineering, broadcasting and TV, women’s union, family planning, academic institutions, transportation, nationality and religion. The role of the committees is to integrate HIV treatment, care, and prevention activities into all government programs. Each department has taken their share of the responsibilities in carrying out the intervention program. In the five years, the Global Fund AIDS Program at provincial and prefecture levels held project meetings, multisectoral coordination meetings, workshops, seminars 965 times. the target of these activities was primarily high-level decision and key policymakers and implementers including legislators, law enforcement personnel, medical professionals, government senior leaders, private sector and civil society leaders including PLHIV, Non-Government Organization (NGO), and Community-Based Organization (CBO) leaders. A total of 1394 projects formed multisectoral agreements were supported by The Global Fund AIDS Program, among which 682 were the government projects and 712 non-government projects. Multistakeholder policy advocacy and support groups (including people living with HIV (PLHIV), community members, and FSWs) were established to determine current priority issues and advocacy messages, and visit project sites in regular base to advocate for improved service delivery and policy implementation. NGOs including Workers’ Union, Youth Union, Red Cross, Science Association, Student Nutrition and Health Promotion Association, Grass Root Organizations, and Self Support Organizations were also mobilized to deliver the treatment, care, prevention services. The capacity of institutional and civil society in planning, management and prevention and care service delivery were strengthened through training, seminars, technical assistance. The government-community partnership was built through improving information and coordination, e.g., the establishment of community advisory boards, a quarterly coordination meeting, and regular information exchange. Quarterly workshops and monthly follow-up meetings were implemented with sex workers, establishment owners and managers, and other establishment employees. The purpose of these encounters was to encourage and strengthen a sense of solidarity and collective commitment toward HIV and STI prevention, care and antiretroviral treatment, and discussion included the role that each actor could play in supporting sex workers to use condoms with their partners. Educational materials were developed and distributed to reinforce responsibilities and benefits pertinent to each group. An additional focus of the workshops and materials was the role of trust and intimacy associated with condom use among sex workers and regular-paying and nonpaying partners.

#### ***Stigma and discrimination reduction at all settings***

Activities including seminars, workshops and publications, and consultation, for stigma and discrimination reduction were organized among service providers, all settings including general public, the workplace. CBOs, NGOs and PLHIV helped to lead anti-stigma trainings for teachers, health care workers, security personnel, and a body of ‘open-source’ supporting materials were collected to be used in the scale-up of similar workshops. In addition, NGOs and CBOs from key partner, sex workers, and health professionals worked with relevant local government departments to advocate and provide training on reducing stigma and mainstreaming anti-discrimination policy and practices into relevant settings, including the health, public security and education sectors, and the private sector.

#### ***100% condom promotion activities***

Multisectoral collaborative condom management, promotion and distribution were implemented systematically. To create an enabling environment for condom and other intervention activities in FSWs, coordination meetings were organized among local health departments, outreach NGOs, police, public security personnel, neighborhood authorities and owners/managers of establishments where FSWs work or gather; experience sharing for establishment owners/managers arranged with other counties where interventions have been successfully been implemented; advocacy for 100% condom use. Free condoms and water-based lubricants were provided to FSWs (particularly street-based FSWs) and other risk groups. Training workshops were held for FSWs on condom use and negotiation. Social marketing of condoms through shops and vending machines was also implemented. Condoms were made easily available through drugstores, small shops, supermarkets, vending machines and at places identified as hotspots for people at high risk, and at drop-in centers, VCT sites, STI clinics and other health facilities. Over three hundred thousand condoms were distributed in six intervention sites.

#### ***Outreach and peer education to promote risk and health-seeking behavior change***

Activities for outreach and peer education included workshops, design/adaptation and pre-testing of materials, materials production and distribution, and evaluation of final products by members of target populations and through field testing. Training and supporting outreach workers and peer educators included: (1) training on outreach and outreach management; (2) training on peer education, (3) conducting outreach work and peer education, (4) local coordination meetings and exchange of experience; (5) revision and updating of the training manual and a variety of guidelines and documentation for used by outreach workers and peer educators, including topics, condom promotion and distribution, promotion of STI services, HIV testing and counseling, local service providers and referral information; 6) collection and reporting of program data. Outreach workers and peer educators included full-time and part-time personnel from NGOs/CBOs (including people from key FSWs), local Center for Disease Control and Prevention (CDC), health centers and other relevant organizations. A comprehensive range of interventions, using traditional and innovative strategies for different settings, were implemented.

#### ***Expand HIV testing and promote standardization of clinical STI services***

A total of 66 voluntary counseling and testing (VCT) sites were established in six intervention sites. Training for staff providing VCT was emphasized how to assess and refer people diagnosed with HIV to other services including antiretroviral treatment, and other care and prevention services. Over thirty thousand participants visited the VCT sites in the program counties. The program provided both HIV testing in VCT sites and designated health facilities under the adapted national technical guidelines, and referring HIV positive clients to local CDCs for follow-up. At the time the intervention began, FSWs were required to attend STI checks at government clinics, and health professionals were mandated to monitor their attendance. Our intervention activities provided clinicians and inspectors with training in the areas of basic HIV/AIDS information, communication skills, reproductive health education, health counseling, data collection and monitoring, and ethical procedures.

#### ***Monitoring and encouraging adherence***

In the six intervention counties, establishment owners were notified of their status in terms of adherence to 5 study elements on a monthly basis: presence of posters, visible condoms, stocks of at least 100 condoms, attendance of sex workers, and lack of positive STI diagnoses among the establishment’s sex workers. These elements were evaluated each month by the inspectors. At the end of each month, establishments that were not adherent these elements were the focus of intensified educational efforts.

#### ***Increase access to high quality of HIV/AIDS treatment, care and support and secondary prevention services for PLHIV***

Activities included (1) adapt and widely disseminate national technical guidelines and protocols on the provision of HIV/AIDS treatment, care and support to PLHIV at the local level; (2) provide training of health care providers, especially grassroots health workers, and including laboratory staff; (3) increase access to and improve quality of ART to PLWHIV in adherence to the adapted national guidelines; (4) provide regular monitoring to ART patients, including clinical and adherence monitoring; (5) train and support peer counselors to provide peer education and support for improving ART adherence; (6) identify, train and support local NGOs, CBOs and relevant government agencies to provide care and support to people living with and affected by HIV/AIDS in collaboration with local CDC and health facilities; (7) provide a continuum of care and support to people living with and affected by HIV/AIDS, including psychosocial support, positive prevention, nutritional support, poverty alleviation; and (8) provide regular clinical monitoring to people living with HIV who are not currently on treatment. There were 328 AIDS patients received ART treatment, 108 children influenced by HIV/AIDS got care and supported. There were a total of 1050 supported activities developed in the six intervention sites.

### Recruitment and participants

Sociodemographic mapping was conducted with venue-based recruitment, community outreach and peer referral techniques to recruit the study participants. A detailed geographical map with roads, key streets, markets, shops, hotels, and bus depots was developed in each county. The numbers of FSWs in each establishment and street were numerated and/or estimated. All establishments and streets were listed and stratified within the sampling frame. Participants were recruited using a convenience sample strategy from randomly selected establishments or streets. A maximum of 25 participants were recruited from the large selected establishments; for smaller selected establishment with ≤15 FSWs. All potential participants in the selected sites were approached and invited for eligibility assessment. Recruitment criteria required that participants be female, willing to participate and complete the survey, and self-reported having had commercial sex in the past month. Verbal informed consent was obtained from all participants. Structured questionnaire-based interviews were conducted by trained experienced health professionals with assistance from trained FSW peers. Voluntary participation, anonymity and confidentiality were ensured for all participants. This study was approved by the Institutional Review Board of Shandong CDC.

### Measures

Questionnaire-based interviews provided demographic information, sexual and drug use behavioral information, ever had HIV testing, HIV knowledge and received HIV-related services in the past year. HIV knowledge was assessed by 8 questions based on correctly identifying modes of HIV transmission and prevention as well as misperception. All questions were equally weighted. Responses to these questions were combined into an overall score for each correct and incorrect answer. These scores were then stratified into 2 groups, according to whether 6 or more appropriate responses were given to the 8 questions. The use of HIV-related prevention service was assessed by 5 indicators related to relevant prevention services received in the last 12 months (ever had a test for HIV, STD examination and/or treatment; or received free condoms, peer education, and HIV-related education materials). The survey instruments were used from the China National Comprehensive Surveillance Survey Guideline, recommended by a panel of national surveillance experts [36]. Serospecimens were collected from all participants. Two screening tests using an enzyme-linked immunoassay (Beijing Jinhao Biologic Production Co. and Beijing Wantai Biological Medicine Company, Beijing, China) were performed to determine HIV serostatus. Syphilis was determined using rapid plasma reagin (RPR™) and a Passive Particle Agglutination Test for Detection of Antibodies to Treponema pallidum (TPPA™, Rong Sheng Biostix Inc, Shanghai, China). These methods were described elsewhere [24].

### Data analysis

Questionnaire-based data were recorded and double-examined with EpiData software (EpiData 3.0 for Windows™, The EpiData Association Odense, Denmark). SPSS® software (Version 15.0; SPSS Inc., Chicago, Illinois, US) was utilized for all analyses. We used data validation tools available in SPSS to check for duplicate data entry. Chi-square test was applied for univariate analysis of variables of sociodemographic and behavioral characteristics, use of HIV-related intervention services, and HIV knowledge score. Multivariable logistic regression models were constructed using a stepwise backward sequence. Both adjusted odds ratios (AOR) and 95% confidence intervals (CI) were obtained for each explanatory variable in the final models. Variables with p < 0.05 in multivariable analysis were considered statistically significant.

## Results

### Characteristics of participants

A total of 3326 FSWs were interviewed in the cross-sectional survey. Of the 3326 participants, 1157 came from the intervention sites and 2169 from control sites. The compositions of samples in intervention sites and control sites were not different by education, over 85% received middle school or higher education and only 12.3% received primary school or lower education in intervention and control sites. The compositions of samples were different by age, marital status, economic conditions and recruitment venues in intervention and control sites. The proportion of subjects aged above 25 years was 31.5% in intervention sites compared 34.2% in control sites; being single accounted for 69.3% in intervention sites compared 66.3% in control sites; 60.9% came from developed area in intervention sites and 54.0% in control sites, 36.5% recruited from clubs/KTV and 14.3% from non-venue based in intervention sites while the proportion were 55.0% and 7.0% in control sites, respectively.

### HIV/syphilis prevalence and sexual/drug use behaviors

No participant was found HIV positive both sites of this study. Syphilis prevalence was 0.17% among the participants in intervention sites versus 1.89% in control sites. In intervention sites, 92.7% of the participants reported condom use in the last sex episode with clients versus 74.7% in control sites. The proportion of consistent condom use with clients in the last month among the participants is significantly higher in intervention sites (77.7%) and control sites (51.7%) (*P* < 0.001). The proportions of participants reported ever having regular sex partners among the participants were similar in intervention sites (41.8%) versus control sites (39.3%) (*P* > 0.05), but the condom use rate in the last sex (67.9%) and consistent use rate in the last month (37.6%) were significantly higher among the participants in the intervention sites than that (54.0%, last sex; 28.9%, consistent use) in the control sites (P < 0.001). There was 0.1% participants reported having ever used illicit drugs in intervention versus 0.8% in control sites (Table 1). Multiple logistic regression models suggested that the rates of condom use in the last episode with clients (AOR = 2.7; 95% CI: 1.9, 3.8), with regular sex partners (AOR = 1.5; 95% CI: 1.1, 1.9) and consistent condom use in the last month with clients (AOR = 3.3; 95% CI: 2.6, 4.1) and regular sex partners (1.7; 95% CI: 1.3, 2.3) were significantly higher in intervention sites than that in control sites (Table 2).

**Table 1:** Demographics, HIV-related knowledge, risk behaviours and HIV intervention service utilization among female sex workers in Shandong Province, China

**Variables**	**Intervention sites (n = 1157)**		**Control sites (n = 2169)**		***P***
	**N**	**%**	**N**	**%**	
**Age (years of age)**					
<20	193	16.7	433	20.0	
20-	600	51.9	996	45.9	0.003
25-	216	18.7	474	21.9	
30-	148	12.8	266	12.3	
**Education**					
Primary school or lower	142	12.3	266	12.3	
Middle school	683	59.3	1296	59.8	0.964
High school or higher	327	28.4	607	28.0	
**Marital status**					
Single	800	69.3	1439	66.3	
Married	177	15.3	513	23.7	
Cohabit	99	8.6	121	5.6	<0.001
Divorce or widow	78	6.8	96	4.4	
**Economic conditions**					
Developed counties	705	60.9	1171	54.0	<0.001
Less developed counties	452	39.1	998	46.0	
**Recruitment venues**					
Sauna	206	17.8	260	12.0	
Clubs or KTV	422	36.5	1192	55.0	
Hotels	137	11.8	204	9.4	<0.001
Hair/beauty salons	227	19.6	359	16.6	
Non-venue based	165	14.3	151	7.0	
**HIV-related knowledge**					
Score <6	193	16.7	1695	78.1	<0.001
Score ≥6	964	83.3	474	21.9	
**Condom use in the last sex episode with clients**					
Yes	1073	92.7	1622	74.7	
No	84	7.3	547	25.2	<0.001
**Condom use with clients in the last month**					
Never	3	0.3	49	2.3	
Sometimes	255	22.0	999	46.1	<0.001
Always	899	77.7	1121	51.7	
**Ever had regular partner**					
Yes	483	41.8	861	39.3	0.188
No	672	58.2	1308	60.7	
**Condom use in the last sex with regular partner**					
Yes	317	67.9	465	54.0	
No	150	32.1	396	46.0	<0.001
**Condom use with regular partner in the last month**					
Never	35	7.3	143	16.6	
Sometimes	265	55.1	471	54.6	<0.001
Always	181	37.6	249	28.9	
**Ever used illicit drugs**					
Yes	1	0.1	17	0.8	
No	1154	99.9	2152	99.2	0.009
**HIV-related intervention services in the last year**					
Ever had a test for HIV					
Yes	798	69.0	729	33.6	
No	359	31.0	1440	66.4	<0.001
STD examination and/or treatment					
Yes	592	51.2	363	16.8	<0.001
No	565	48.8	1799	83.2	
Free condom					
Yes	1096	94.7	1237	57.1	<0.001
No	61	5.3	931	42.9	
Peer education					
Yes	745	64.4	581	26.8	<0.001
No	412	35.6	1587	73.2	
HIV education materials					
Yes	1124	97.1	1384	63.8	
No	33	2.9	784	36.2	<0.001
**Sought medical treatment when your body condition has some disorder**					
Yes	1004	86.8	1451	66.9	<0.001
No	153	13.2	718	33.1	

* Sub-groups do not always add up to totals due to missing data.

**Table 2:** HIV knowledge, condom use, HIV-related intervention services, and sought medical treatment behavior compared to intervention sites to control sites in Shandong Province, China

**Factors**	**OR (95% CI)**	AOR* (95% CI) ^**†**^
**HIV knowledge**		
Score ≥6	17.9 (14.8, 21.5)^¶^	24.7 (2.5, 42.7)^¶^
**Condom use in the last sex episode**		
With clients	2.4 (1.9, 3.1)^¶^	2.7 (1.9, 3.8)^¶^
With regular sex partners	1.8 (1.4, 2.3)^¶^	1.5 (1.1, 1.9)^§^
**Consistent condom use in the last month**		
With clients	3.3 (2.7, 3.8)^¶^	3.3 (2.6, 4.1)^¶^
With regular sex partners	1.4 (1.2, 1.8)^§^	1.7 (1.3, 2.3)^¶^
**Received HIV-related services in the last year**		
HIV Testing	4.8 (2.9, 6.1)^¶^	4.9 (2.8, 6.7)^¶^
STD examination and/or treatment	5.2 (4.4, 6.1)^¶^	5.1 (4.2, 6.4)^¶^
Free condom	13.5 (10.3, 17.7)^¶^	20.3 (14.3, 28.9)^¶^
Peer education	4.9 (4.2, 5.8)^¶^	4.3 (3.5, 5.4)^¶^
HIV-related education material	19.3 (13.5, 27.6)^¶^	19.8 (13.1, 29.8)^¶^
**Sought medical treatment when your body condition has some disorder**	3.2 (2.7, 3.9) ^¶^	3.2 (2.5, 4.2)^¶^

*AOR: adjusted odds ratio. All of these AORs were adjusted for age, marital status, education, economic condition, recruitment venues; ^†^CI: confidence interval; §: P < 0.01; ^¶^: P < 0.001; NS: no statistical significance; STD: Sexually transmitted diseases.

### HIV-related knowledge and HIV-related service utilization

After adjusted for age, marital status, education, economic condition, recruitment venues, the proportion of participants correctly answered at least six out of eight HIV-related questions (83.3%) in intervention sites is significant higher than that (21.9%) in control sites (AOR = 24.7; 95% CI: 2.5, 42.7) (Tables 1 and 2), the five indicators related to HIV-related services ever received in the last year including HIV testing (AOR = 4.9; 95%CI: 2.8, 6.7), STD examination and/or treatment(AOR = 5.1; 95% CI: 4.2, 6.4), free condom (AOR = 20.3; 95% CI: 14.3, 28.9), peer education(AOR = 4.3; 95% CI: 3.5, 5.4), HIV-related education material (AOR = 19.8; 95%CI: 13.1, 29.8) were significantly higher in intervention sites than that in control sites (Table 2), the participants in the intervention sites are more likely to seek medical treatment when they had any disorders (AOR = 3.2; 95% CI: 2.5, 4.2).

## Discussion

HIV interventions focused on high risk groups, e.g., FSWs, have often targeted individual behavior, with impediments to success frequently including structural factors beyond the scope of the programs [17,37,38]. In this study, we have examined the integrated individual, community, structural intervention to HIV/STI prevention, which sought to change those context-specific political, social, legal, and environmental factors that known to affect HIV/STI risk and vulnerability [16,17], as part of a large-scale Comprehensive AIDS Response, community-based HIV treatment, care, prevention program, in Shandong Province.

This program focused on addressing structural factors using multisectoral collaborative strategies, following individual level intervention and community mobilization of FSWs, worked in collaborative partnership with policy makers, multisectorial stakeholders and civil society (including CBOs/NGOs and FSWs themselves) to address structural barriers, stigma and discrimination facing the FSW community.

The findings of this study revealed the significant lower rate of syphilis, higher score of HIV knowledge, and the higher rates of condom use, HIV testing and the utilization of HIV prevention services in the intervention sites, compared to that in the control sites, which suggested the successes of the integrated individual, community and structural intervention. These findings are consistent with our previous findings [39] and which also support the finding of other studies in other Asian country that have showed significant increases in condom use, reductions in HIV and STI rates among FSWs [40-42].

The higher score of HIV knowledge among FSWs demonstrated in intervention sites is consistent with our previous study [39]. Our previous study targeted FSWs in the same 6 sites from 2004 to 2008 showed the rate of correct answer of three HIV/AIDS transmission routes increased from 59.36% at baseline in 2004 to 97.21% in 2008, and increased from 32.76% in 2004 to 82.76% in 2008 for correct answer of non-transmission routes. The higher rate of condom use found in the intervention sites is also consistent with our previous report that the proportion of consistent condom use with clients in the last three months significantly increased from 38.75% at baseline in 2004 to 71.67% in 2009 [39]. In addition, the overall low HIV prevalence indicated from surveillance system and lower syphilis prevalence in intervention sites compared to the control sites indicated the integrated individual level intervention, community mobilization and structural intervention prevention program with multi-sectoral cooperation and strong societal participation made the difference.

Although the progress was evidenced by the increase in condom use in intervention sites compared with control sites, the rates of condom use among FSWs are still low. Condom use has been proven to effectively prevention the transmission and control the epidemic among FSWs, but the coverage of condom is the key [27,43-47]. The Asian Epidemic Model predicts that a high rate of condom use (>85%) among FSWs is required to control the HIV epidemic [27,43,46]. Data also showed high levels of condom use among FSWs and their clients in countries such as Benin, Thailand and Cambodia are likely to have slowed the epidemic [10,48]. Shandong with 140 counties is the second most populous province in China. The study indicated that the progress on HIV/STD intervention has been made uneven among counties across the province and within counties. This study also showed two-thirds of them had not previously tested for HIV. The relative low condom use and utilization of HIV-related prevention services in both intervention and control sites highlighted the needs of continued effort in scaling up the care, treatment and prevention activities.

Commercial sex in China is illegal. FSWs are often blamed for the breakdown of the traditional family, epidemics of STIs and HIV/AIDS, and escalating crime [22,24]. Therefore, stigma and discrimination and other potential barriers might have prevented FSWs seeking VCT or other HIV prevention and care services for fear of discrimination, fear of disclosure of commercial sex activities, low of confidentiality [24,49], and fear of the punishment of the illegal commercial sex. Study also showed stigma and discrimination, site atmosphere, links with other services, quality of the staff, and convenience were important factors for FSWs actively receiving HIV testing and HIV-related services [50,51]. Hence, the stigma and discrimination reduction effort with multi-sectoral cooperation and strong societal participation, years of efforts of intervention service promotion, well trained health professionals, peer education, condom distribution and promotion, should be a critical part of the large scale Comprehensive AIDS Response program to contain STIs and HIV/AIDS epidemic among FSWs.

Trainings on discrimination and stigma have been a part of China’s response to HIV for many years, but there remains a high degree of ignorance, misinformation and stigma surrounding the virus [52]. This is slowly changing, and a growing number of PLHIV are now willing, given the proper support, to reveal their status. It is therefore now possible to adjust the strategy and adopt a new method: engaging HIV infected individuals and encouraging them to participate in all aspects of anti-stigma campaigns. The fundamental logic behind this activity is that PLHIV involvement in anti-stigma activities can greatly improve their effectiveness [52]. The stigmatization of HIV-infected people and many of the sub-populations who are most vulnerable to infection, and the discrimination that results from it, act as constraints on the delivery of prevention and health care services and damage the lives of those infected with or affected by HIV/AIDS. A high degree of stigma also helps to keep people ignorant about the disease, which increases their own risk of becoming infected. Tackling discrimination and stigma was therefore an integral part of this integrated intervention. Efforts were informed by international best practices related to reducing stigma and discrimination and advocacy for the rights of PLHIV and other key affected populations. By reducing stigma and discrimination in all settings and particularly in health care settings, it is expected that more of the people who are infected or are most at risk of infection will be encouraged to access prevention services, treatment, care and support. Although no data were collected in evaluating the changes of stigma and discriminatory practices and attitudes, the efforts in reducing stigma and discrimination likely affected the behavior change towards eliminating attitudes and practices that act as barriers to ensuring the rights of PLHIV and key affected populations and their access to services and entitlements. The higher rates of HIV testing and the utilization of HIV prevention services in intervention sites, compared to the control sites, which may reflect certain success of stigma and discrimination reduction effort.

The effective method of promoting multisectoral participation in HIV/AIDS prevention, care and treatment is to ensure that each sector has its own responsibilities, in line with its respective strengths and comparative advantages, under an agreed HIV/AIDS action framework. The involvement of community such as CBOs, grassroots NGOs and organizations representing PLHIV and FSWs, is encouraged by the Global Fund AIDS Program, has played a consistently important role in increasing capacity building and technical support to civil society. Effective multisectorial cooperation and improved community participation in the six intervention sites ensured the successful implementation of the community-based HIV treatment, care and prevention program.

Strengths of this study include its estimation of the size of the targeted population and mapping strategy. Efforts on investigating the venue and preliminary estimates in each establishment strengthen the mapping strategy and targeted population size estimation to reduce selection bias. Our study subjects may represent a wider spectrum of FSWs in Shandong. The socio-demographic and behavioral factors identified in our study are informative, giving us a stronger grasp of Shandong’s current HIV epidemic and risk behavior and the use of prevention services among FSWs. We also recognized the limitations of this study. Selection bias existed in this study. Non-response information was not collected. The questionnaire data relying on retrospective self-reports was subject to recall bias. Sensitivity of sexually-related questions could lead to reporting bias. The cross-sectional research design precludes identification of causal relationships. No baseline survey conducted among control sites could be another limitation of the study. This study also served as a behavior surveillance survey, which was conducted with careful planning, implementation, and quality control. In spite of these limitations, we believe that this study provide invaluable information to guide the ongoing comprehensive community-based treatment, care, prevention intervention program among this group.

## Conclusion

In summary, the results of the study indicated that significant progress has been made in the intervention sites compared with control sites among FSWs. This study demonstrated that the integrated individual, community, and structural intervention have positive impact in reducing HIV and STI risks among FSWs. The findings highlighted the needs of effective multisectoral cooperation with broad community participation in implementing the comprehensive community based HIV treatment, care and prevention program. Structural factors including stigma and discrimination are critical concerns among this FSW population and needed to be addressed to enable vulnerable women to adopt the safer sexual behaviors that are required to respond the HIV/STI epidemic.

## Abbreviations

FSWs: Female commercial sex workers; HIV: Human immunodeficiency virus; STI: Sexual transmitted infection; STD: Sexual transmitted disease; PLHIV: People living with HIV; CBO: Community based organization; NGO: Non-government organization; VCT: Voluntary counseling and testing; CDC: Center for disease control and prevention; ART: Antiretroviral treatment; AIDS: Acquired immunodeficiency syndrome; CI: Confidence interval; AOR: Adjusted odds ratio.

## Competing interests

The authors declare that they have no competing interests.

## Authors’ contributions

DK, XT, ML, and YJ contributed to the study design and literature searches and drafted the manuscript. ML, JL, XZ and NZ performed all the statistical analyses. SS, BL, and XS supervised laboratory test. DK and XT helped to draft the manuscript and critically reviewed the manuscript. All authors read and approved the final version of the manuscript.

## Pre-publication history

The pre-publication history for this paper can be accessed here:

http://www.biomedcentral.com/1471-2458/13/717/prepub
